# Src-1 and SP2 promote the proliferation and epithelial–mesenchymal transition of nasopharyngeal carcinoma

**DOI:** 10.1515/med-2021-0248

**Published:** 2021-07-15

**Authors:** Jingjing Zhang, Yuanyuan Yang, Hongyu Liu, Hongyi Hu

**Affiliations:** Ear, Nose, Throat Department, Peking University Shenzhen Hospital, Shenzhen, Guangdong 518000, China

**Keywords:** nasopharyngeal carcinoma, steroid receptor coactivator 1, SP2, epithelial–mesenchymal transition

## Abstract

Nasopharyngeal carcinoma (NPC) is characterized by high morbidity and morality, especially in Southern China. Transcription factors intensively participate in the initiation and development of NPC. This study aimed to investigate the roles of Src-1 in NPC. mRNA level was determined by qRT-PCR. Western blot was carried out for the protein level. CCK-8 assay was performed to determine cell viability, colony formation for NPC cell proliferation, and transwell for cell migration and invasion ability. The results showed Steroid receptor coactivator 1 (Src-1) was overexpressed in SNE-2 and 6-10B. The expression of Src-1 and SP2 was in positive correlation. Overexpression of Src-1 promoted the cell viability, colony formation, and epithelial–mesenchymal transition (EMT), manifested by the increase of migration and invasion ability, while knockdown of Src-1 exerted opposite effects. Additionally, knockdown or overexpression of SP2 reversed the effects of overexpressed or downregulated Src-1, which was reversed by the depletion of SP2. Moreover, Src-1 interacted with SP2 to regulate EMT-related genes such as E-cad, N-cad, Vimentin, and ZEB1, and proliferation- and apoptosis-related genes, such as bax, cytochrome c, and cleaved caspase3 and bcl-2. Thus, blocking the interaction between Src-1 and SP2 may be a therapeutic target for inhibiting the metastasis of NPC.

## 1 Introduction

Nasopharyngeal carcinoma (NPC) is originated from the epithelium of nasopharynx [1]. Nowadays, the incidence of NPC is highest in Southern China and Southeast Asia [2]. Almost four-fifths of all NPC cases are in Asia and most of the cases are diagnosed at advanced stage [3]. Various factors induce the initiation and progression of NPC [4]. Although great breakthroughs have been made in the therapy of NPC in the past decades, the morbidity of NPC remains high and the clinical results in many cases are still unsatisfactory due to the progressive recurrence of NPC and distant metastasis [5,6]. Distant metastasis is the main cause for NPC-related death [7]. To explore the potential mechanisms underlying the progression of NPC may provide an effective therapy for NPC.

Tumor metastasis is a complicated process, including local invasion, intravasation, dissemination, extravasation, and colonization [8,9]. epithelial–mesenchymal transition (EMT) is a crucial factor for the acquired metastatic ability of tumor cells [10,11]. EMT is accompanied with the degradation of epithelial features and the acquisition of invasive mesenchymal phenotype [12]. Therefore, EMT is paralleled with the degradation of epithelial cell adhesion molecules and the increase of mesenchymal markers [13]. Interestingly, EMT is modulated by transcription factors (TFs), such as ZEB, Snail, and Twist. The activation of TFs binds to its targets to modulate epithelial or mesenchymal traits, contributing to the progression of cancer. Steroid receptor coactivator 1 (Src-1 or NCOA1) is a promoter of progesterone receptor [14]. Additionally, Src-1 works with other transcriptional factors such as TTF-1 and PEA3 [15,16]. Src-1 interacts with other TF or regulates specific pathways to modulate cell proliferation, apoptosis and EMT, etc. [17,18,19]. Increasing evidences demonstrate Src-1 functions as an oncogene in cancer, such as breast cancer, thyroid cancer, as well as NPC [19,20,21]. However, the potential roles of Src-1 in NPC have not been fully elucidated.

SP families (SP1–9) participate in various biological processes via regulating its downstream [22]. Dysregulated SPs are associated with various disease, including NPC. SP1 and SP3 promote the development of NPC via binding to centromere protein H [23]. However, the potential roles of SP2 in cell and organismal physiology have not been fully elucidated. SP2 is widely expressed and performed weakly only if promoters are with consensus SP2-binding sites or well-characterized by SP1 and SP3. SP2 regulate the promoter of gene associated with cell cycle, proliferation, inflammatory response, invasion, metastasis, and EMT. The abnormal levels of SP2 induce the aggressive phenotype of hepatocellular carcinoma, gastric cancer, and pancreatic cancer [24,25,26]. However, the potential roles of SP2 in NPC is still unclear.

In the study, Src-1 and SP2 were upregulated in NPC cells. Meanwhile, Src-1 interacted with SP2 to promote the proliferation and EMT of NPC. Thus, Src-1 may be a potential biomarker for NPC.

## 2 Materials and methods

### 2.1 Cell culture

Human NPC cell lines (CNE1, SNE-2, 5-8F, and 6-10B) and nasopharyngeal epithelial cell line NP69 cells were obtained from American type culture collection (ATCC), USA. Cell were incubated in RPMI-1640 containing 10% FBS at 37℃ under 5% CO_2_.

### 2.2 Transfection

Src-1 overexpression plasmids (Src-1), Src-1 knockdown plasmids (si-Src-1), SP2 overexpression plasmids (SP2), SP2 knockdown plasmids (si-SP2), and the empty vector (Vector), SP2 were purchased from GenePharm, Shanghai. SNE-2 and 6-10B were treated with Src-1, si-SP2, or Vector with Lipofectamine 2000 (Invitrogen, USA). After 48 h, the cells were used in the following experiment.

### 2.3 qRT-PCR

Total RNA was separated from cells. RNA was synthesized into cDNA using a cDNA reverse transcription kit (Takara, Japan). PCR was conducted with SYBR^®^ Green PCR Master mix (Applied Biosystems, USA) on ABI 7500 Real-Time PCR System under the following thermocycling conditions: 50°C for 2 min, and denaturation at 95°C for 10 min, followed by 40 cycles of 95°C for 15 s and 60°C for 1 min. GAPDH served as loading control. Each experiment was performed thrice. The sequences of the primer used were as followed: Src-1: Forward: 3′-TCACTTCAGTCCGCCACT-5′; Reverse: 3′-TCGCCTGTTCCTGGTTGT-5′; SP2: Forward: 5′-CCAGCCTACCCCAAGGAAAC-3′; Reverse: 5′-GGGAGCCCTGAATCTGAAGTAT-3; GAPDH: Forward, 5′-CGGAGTCAACGGATTTGGTCGTAT-3′; Reverse, 5′-AGCCTTCTCCATGGTGGTGAAGAC-3.

### 2.4 Western blot

Total protein was collected from cells. The protein concentration was determined by BCA Kit (Beyotime, Shanghai). 20 µg of protein was isolated by 12% SDS-PAGE. Then the separated protein was transferred onto PVDF membranes. Afterwards, the membrane was first incubated with anti-E-cad, anti-N-cad, anti-Vimentin, anti-ZEB1, anti-bax, anti-bcl-2, and anti-cytochrome c, and anti-cleaved-caspase3 at 4℃ overnight in shade. Next day, the membranes were incubated with goat-anti-rabbit at room temperature for 2 h. Finally, the protein bands were captured with a ECL kit on image-pro plus software 6.0.

#### 2.4.1 CCK-8

The cell viability was determined with a CCK-8 Kit (Beyotime, Shanghai). SNE-2 and 6-10B were planted in 96-well plate. Then each plate was added with 20 μL CCK-8 solution. The absorbance of the cells for 0, 24, 48, and 72 h at the wavelength of 450 nm with a microplate reader.

### 2.5 Colony formation assay

Cells were cultured with a 96-well plate. After 12-day cultivation, each plate was fixed with 4% paraformaldehyde and then stained with a 0.4% crystal violet. Subsequently, the colonies were captured using a dissection microscope and calculated.

### 2.6 Transwell

Cells were planted into a 24-well plate and incubated in the upper chamber with or without Matrigel. The bottom chamber was treated with 10% FBS. At 24 h, the non-migrated or invaded cells were removed. The migrated or invaded cells were added with 4% methanol and crystal violet. Finally, the cells were captured and counted.

### 2.7 Statistical analysis

All data were represented as mean ± SD and analyzed with SPSS 19.0. The difference was analyzed with one-way ANOVA. *P* < 0.05 was deemed as statistical significance.

## 3 Results

### 3.1 The expression of Src-1 is increased in NPC cells

**Figure 1:**
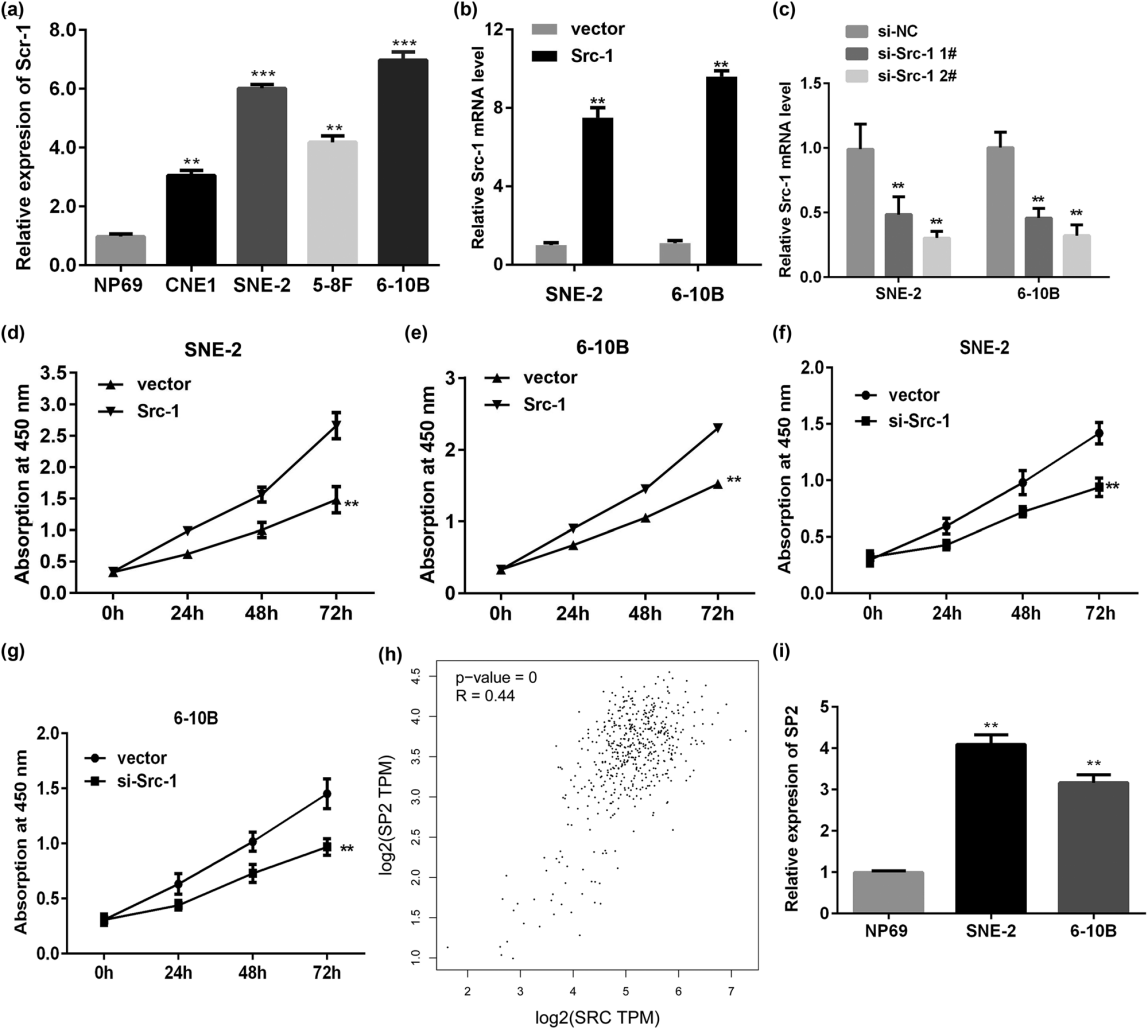
(a) The mRNA level was significantly increased in NPC cells, especially in SNE-2 and 6-10B. (b,c) The transfection efficiency was determined by qRT-PCR. (d,e) Overexpression of Src-1 increased the cell viability of 6-10B. (e) Knockdown of Src-1 suppressed the cell viability of SNE-2. (f,g) Knockdown of Src-1 suppressed the cell viability of SNE-2. (h) EPIA analysis showed the expression of Src-1 and SP2 was in positive correlation. (i) SP2 was upregulation in NPC cells. **P < 0.01, ***P < 0.001 vs. NP69 or vector group.

qRT-PCR was conducted to detect the level of Src-1 in NPC. As showed in Figure 1a, Src-1 was significantly upregulated in NPC cells in comparison with normal cells. Moreover, the expression of Src-1 in SNE-2 and 6-10B was more remarkable. Therefore, Src-1 in SNE-2 and 6-10B was applied in the following experiment. After transfected with Src-1 overexpression plasmids, the level of Src-1 was significantly increased (Figure 1b). Additionally, the expression of Src-1 was downregulated in cells transfected with si-Src-1, which was more potent in si-Src-1 2# group (Figure 1c). Hence, si-Src-1 2# was used in the following experiment. Overexpression of Src-1 remarkably promoted the cell viability of SNE-2 and 6-10B compared with empty group (Figure 1d and e). Knockdown of Src-1 significantly suppressed the cell viability of NPC cells (Figure 1f and g). Moreover, to investigate the underlying molecular mechanisms, we examined the downstream of Src-1. As showed in Figure 1h, the online database GEPIA showed that the expression of Src-1 was in positive correlation with SP2. Then we determined the expression level of SP2 in NPC cells. As showed in Figure 1i, SP2 was significantly upregulated in NPC cells.

### 3.2 Overexpression of Src-1 facilitates the cell viability and proliferation of NPC cells via regulating SP2

**Figure 2:**
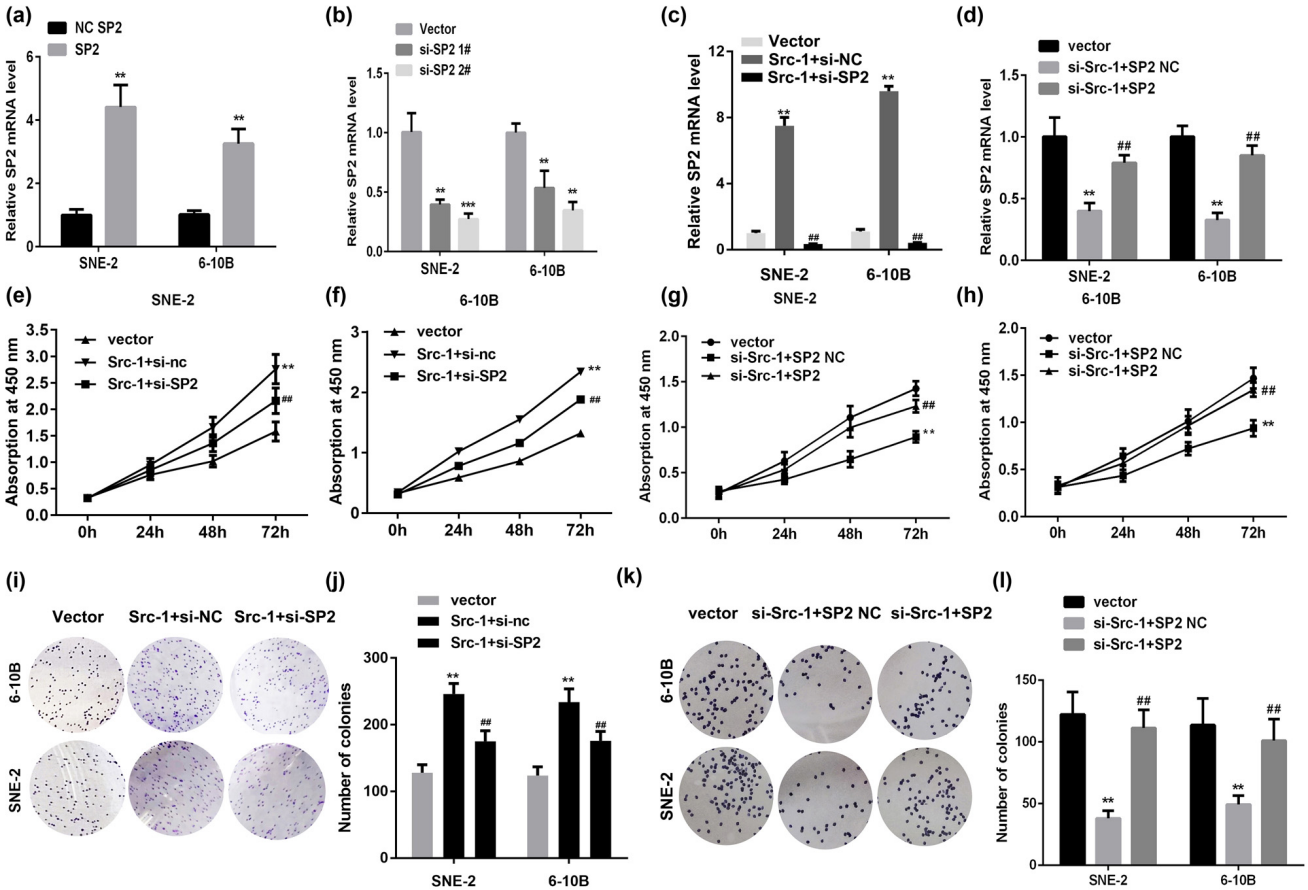
Src-1 facilitates the cell viability and proliferation of NPC cells via regulating SP2. (a) The expression of SP2 determined by qRT-PCR. (b) The mRNA level of SP2 detected by qRT-PCR. (c) The expression of SP2 determined by qRT-PCR. (d) The mRNA level of SP2 detected by qRT-PCR. (e–h) Cell viability of NPC cells detected by CCK-8. (i–l) The proliferation of NPCs determined by colony formation assay. **P < 0.01 vs Vector group. ^##^ *P* < 0.01. vs Src-1 + si-NC group or si-Src-1 + SP2 NC group.

To verify the potential roles of Src-1 in NPC, we investigated the roles of Src-1 in the proliferation of NPC. As showed in Figure 2a and c, the expression level of SP2 was upregulated by SP2 overexpression plasmids and decreased by silenced SP2 (Figure 2a and b). Moreover, the upregulation of SP2 induced by Src-1 was reversed by the transfection of SP2 knockdown, while overexpression of SP2 antagonized the downregulation of SP2 induced by si-Src-1 (Figure 2c and d). Moreover, As showed in Figure 2e–h, SP2 knockdown reversed the increase of NPC cell viability induced by overexpressed Src-1, while decrease of NPC induced by si-Src-1 cell viability was reversed by SP2. This was paralleled with the results from the colony formation assay (Figure 2i–l).

### 3.3 Src-1 promotes the migration and invasion ability of NPC cells via regulating SP2

**Figure 3:**
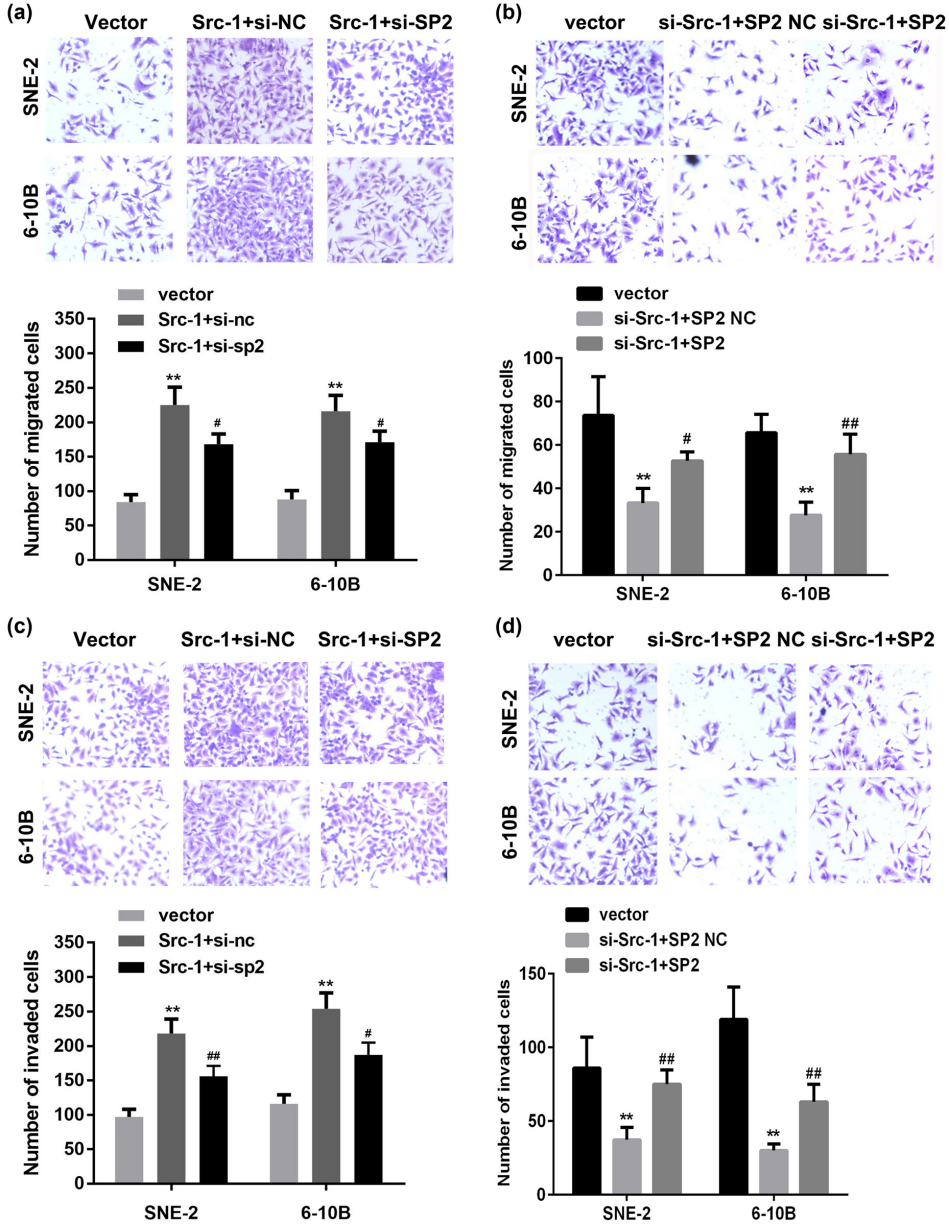
(a,b) The migration ability of NPC cells determined by transwell assay. (c,d) The invasion ability of NPC cells detected by transwell assay. **P < 0.01 vs. Vector group. ^#^ *P* < 0.05, ^##^ *P* < 0.01. vs. Src-1 + si-nc group or si-Src-1 + SP2 NC group.

To further explore the roles of Src-1 in NPC, we evaluated the effects of Src-1 on the migration and invasion ability of NPC cells. Transwell assay was conducted to determine the migration and invasion ability of SNE-2 and 6-10B cells. As showed in Figure 3a, the number of migrated cells in Src-1-treated group was significantly increased in comparison with empty vector group, which was abated by SP2 knockdown. Downregulation of Src-1 significantly inhibited the migration of NPC cells, while overexpressed SP2 reversed this (Figure 3b). Moreover, the overexpression of Src-1 significantly augmented the number of invaded cells; however, downregulation of SP2 antagonized increase of invasion ability of SNE-2 and 6-10B cells induced by upregulation of Src-1 (Figure 3c). Furthermore, overexpressed SP2 abated the inhibitory effects of Src-1 knockdown on the invasion ability of NPC cells (Figure 3d). These results suggested overexpressed Src-1 may enhance the migration and invasion ability of NPC cells.

### 3.4 Src-1 regulates the expression of EMT- and proliferation-related genes in SP2-dependent manner

**Figure 4:**
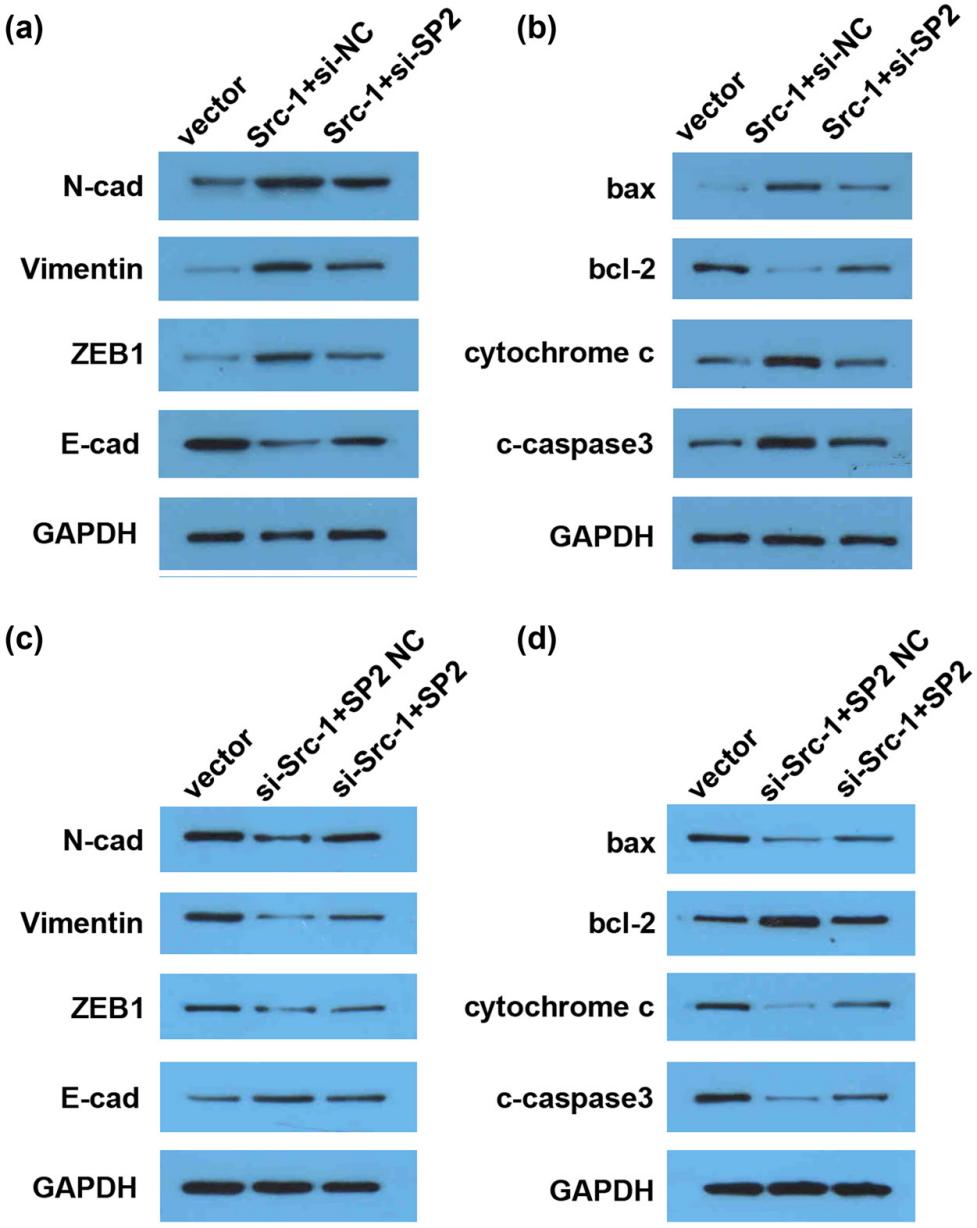
Src-1 regulates the expression of EMT-and proliferation-related genes in SP2-dependent manner. (a–d) The protein level determined by Western blot. Downregulation of SP2 alleviated Src-1-induced decrease of E-cad, bax, cytochrome c, and cleaved caspase3, and increase of N-cad, Vimentin, ZEB1, and bcl-2. Overexpression of SP2 abated the effects of Src-1 knockdown on the expression of E-cad, bax, cytochrome c, and cleaved caspase3, and increase of N-cad, Vimentin, ZEB1, and bcl-2.

Western blot was conducted to determine the protein level. E-cadherin, N-cadherin, and Vimentin are crucial regulators of EMT. Cytochrome c, caspase3, the ratio of bax/bcl-2 is closely associated with cell proliferation and apoptosis. Thus, the effects of overexpressed or knockout Src-1 on the expression of EMT- and apoptosis-related pathways may verify the roles of Src-1 in the proliferation and EMT of NPC. As showed in Figure 4a, Src-1 downregulated E-cad and upregulated N-cad, Vimentin, and ZEB1; the regulatory role of Src-1 was alleviated by downregulation of SP2. Moreover, the overexpression of Src-1 induced the overexpression of bax, cytochrome c, and cleaved caspase3 and the downregulation of bcl-2, which was reversed by SP2 knockdown. However, knockdown of Src-1 had the opposite effects, which was reversed by overexpressed SP2 (Figure 4c and d). These results suggested that the overexpression of Src-1/SP2 interaction promotes the proliferation and EMT of NPC.

## 4 Discussion

NPC ranks as the eighth most common malignancy and is one of the main reasons for cancer-related death [1]. Src-1 has been associated with many types of cancer [15,19,20,21]. However, the potential roles of Src-1 in NPC are still unclear. In this study, Src-1 was upregulated in NPC cells. Moreover, overexpression of Src-1 promoted proliferation, EMT of NPC cells, which was reversed by knockdown of its downstream gene, SP2. Therefore, these results suggested that activation of Src-1/SP2 axis may promote the progression of NPC via enhancing the proliferation and EMT of NPC cells.

Steroid receptor coactivator is “master regulators” of TFs modulating the biological processes in cancer [27]. The potential roles of SRCs in cancer have attracted increasing attention. The activation of SRC in cancer predicts poor clinical results [28,29,30]. As a crucial member of SRCs family, Src-1 promotes the progression of endocrine-resistant breast cancer and resistance to chemotherapeutics [14]. Src-1 binding to NF-kB modulates the level of VEGF to promote the progression of human thyroid cancer [15]. In NPC, Src-1 is overexpressed in NPC tissues in *ex vivo* assay and predicts the unsatisfactory prognosis [19]. Moreover, in this study, Src-1 was upregulated in NPC cells. Moreover, overexpression of Src-1 promoted the viability of NPC cells; however, knockdown of Src-1 suppressed the proliferation of NPC cells. These results suggested that Src-1 may act as an oncogene in NPC, which is consistent with Zhou et al.’s study [19]. However, the underlying molecular mechanisms are still not clear.

The key roles of Srcs in AR transcriptional activity, cell proliferation, migration, and resistance to androgen deprivation therapy in cancer indicate that Srcs may be important therapeutic targets [17,18,19]. A previous study reveals that natural compounds, gossypol and bufalin, possessing Src-inhibitory properties can be novel chemotherapeutics for acquired cancer cell resistance [31]. Additionally, an increasing body of reports focuses on blocking the interaction between Srcs and its downstream. For instance, blocking the interaction between integrin α 5 (ITGA5) and Src-1 antagonized Src-1-induced metastasis of breast cancer [32]. Knockdown of Hepatocyte nuclear factor 4alpha (HNF4alpha) activators, such as PGC1alpha, Src-1, and Src-2, promotes the dedifferentiation of human hepatomas [33]. The interaction between Src-1 and Twist1 promotes the progression of NPC [19]. Thus, Src-1 may be a promising therapeutic target for NPC. In this study, SP2 was upregulated in NPC cells. Moreover, Src-1 was in positive correlation with SP2. Specific proteins (SPs) are the members of SP/Kruppel-like factor (KLF) transcription factor family [26]. SPs regulate a pool of gene expression via modulating the activity of gene promoters [34]. Increasing literatures reveal that SPs function as an oncogene in cancer [26,35]. SP1-induced upregulation of MyD88 contributes to the chemoresistance of breast cancer [26]. SP2 targets TRIB3 to facilitate the migration and invasion ability of hepatocellular carcinoma [36]. Interestingly, SP1 interacts with c-Myc to modulate BMI1 transcription and promotes the progression of NPC [37]. However, the roles of SP2 in NPC have not been elucidated. Thus, we speculated that the interaction between Src-1 and SP2 in NPC may be associated with the progression of NPC. In this study, the expression of Src-1 was positively correlated with SP2. Moreover, Src-1 modulated the proliferation of NPC cells via regulating SP2. Thence, Src-1 may promote the development of NPC in SP2-dependent manner.

Distant metastasis is the key reason for clinical failure. In tumor scenarios, epithelial–mesenchymal transformation (EMT) is an essential step during tumor cell metastasis and differentiation [15]. Numerous evidences demonstrate that transcriptional factors modulate the initiation and development of cancer via regulating various biological processes [27,28,29,30]. Furthermore, it may also work with other transcription factors. YY1 inhibits c-Myc transcriptional activity to inhibit the cell proliferation and cell cycle in NPC [38]. In this study, Src-1 and SP2 may function as oncogenes in NPC. Src-1 interacted with SP2 to modulate the proliferation and EMT of NPC cells. However, blocking the interaction with Src-1 and SP2 exerted inhibitory effects on the progression of NPC. Thence, SP2/Src-1 axis may be a potential target for NPC.

To our knowledge, this is the first study to investigate the roles of Src-1/SP2 axis in NPC. However, there are several limitations in this study. First, the present study was concerned with *in vitro* assay; *in vivo* assay is needed in further study. Second, clinical practice may make the study more convincing.

In conclusion, Src-1 and SP2 were upregulated in NPC. Blocking the interaction between Src-1 and SP2 inhibited the proliferation and EMT of NPC cells. The Src-1/SP2 axis may be a potential therapeutic target for NPC.
